# Identification of Lipocalin 2 as a Ferroptosis-Related Key Gene Associated with Hypoxic-Ischemic Brain Damage via STAT3/NF-κB Signaling Pathway

**DOI:** 10.3390/antiox12010186

**Published:** 2023-01-12

**Authors:** Lianxiang Luo, Liyan Deng, Yongtong Chen, Rui Ding, Xiaoling Li

**Affiliations:** 1 The Marine Biomedical Research Institute, Guangdong Medical University, Zhanjiang 524023, China; 2 The Marine Biomedical Research Institute of Guangdong Zhanjiang, Zhanjiang 524023, China; 3 The First Clinical College, Guangdong Medical University, Zhanjiang 524023, China; 4 Animal Experiment Center, Guangdong Medical University, Zhanjiang 524023, China

**Keywords:** ferroptosis, hypoxic-ischemic brain damage, lipocalin 2, mitochondrial, NF-κB, STAT3, machine learning, WGCNA, PPI

## Abstract

Hypoxic-ischemic brain damage (HIBD) is a common cause of death or mental retardation in newborns. Ferroptosis is a novel form of iron-dependent cell death driven by lipid peroxidation, and recent studies have confirmed that ferroptosis plays an important role in the development of HIBD. However, HIBD ferroptosis-related biomarkers remain to be discovered. An artificial neural network (ANN) was established base on differentially expressed genes (DEGs) related to HIBD and ferroptosis and validated by external dataset. The protein–protein interaction (PPI) network, support vector machine-recursive feature elimination (SVM-RFE) algorithms, and random forest (RF) algorithm were utilized to identify core genes of HIBD. An in vitro model of glutamate-stimulated HT22 cell HIBD was constructed, and glutamate-induced ferroptosis and mitochondrial structure and function in HT22 cells were examined by propidium iodide (PI) staining, flow cytometry, Fe^2+^ assay, Western blot, JC-1 kit, and transmission electron microscopy (TEM). In addition, Western blot and immunofluorescence assays were used to detect the NF-κB/STAT3 pathway. An HIBD classification model was constructed and presented excellent performance. The PPI network and two machine learning algorithms indicated two hub genes in HIBD. Lipocalin 2 (LCN2) was the core gene correlated with the risk of HIBD according to the results of differential expression analysis and logistic regression diagnostics. Subsequently, we verified in an in vitro model that LCN2 is highly expressed in glutamate-induced ferroptosis in HT22 cells. More importantly, LCN2 silencing significantly inhibited glutamate-stimulated ferroptosis in HT22 cells. We also found that glutamate-stimulated HT22 cells produced mitochondrial dysfunction. Furthermore, in vitro experiments confirmed that NF-κB and STAT3 were activated and that silencing LCN2 could have the effect of inhibiting their activation. In short, our findings reveal a molecular mechanism by which LCN2 may promote ferroptosis in HIBD through activation of the NF-κB/STAT3 pathway, providing new and unique insights into LCN2 as a biomarker for HIBD and suggesting new preventive and therapeutic strategies for HIBD.

## 1. Introduction

Hypoxic-ischemia (HI) injury remains one of the most common causes of brain damage in newborns. Neonatal HIBD is a condition in which fetal and neonatal brain dysfunction is caused by hypoxia and reduced or suspended cerebral blood flow due to perinatal asphyxia, and it is one of the neurodegenerative diseases. Therapeutic hypothermia (TH) is currently the standard treatment for HIBD, and its ability to improve neuronal death in the prognosis of HIBD may reduce the risk of cerebral palsy and severe disability in children with moderate to severe hypoxic-ischemic encephalopathy (HIE) [1,2]. However, despite treatment with TH, surviving neonates are still at risk of cognitive impairment, learning disabilities, and developmental delays, placing a heavy burden on families and society. The exact pathogenesis of HIBD is still not fully understood; therefore, there is an urgent need to elucidate the pathogenesis of neonatal HIBD to further improve the prognosis of neonatal HIBD patients.

Ferroptosis is an iron-dependent form of regulated cell death that is triggered by the toxic build-up of lipid peroxides on cellular membranes [3]. There is now growing evidence that ferroptosis is involved in the pathogenesis of central nervous system (CNS) diseases, including HIBD, and that inhibition of ferroptosis may prevent neuronal death in some CNS diseases [4,5]. Neonatal rats undergo a series of oxidative stress responses after experiencing hypoxia and ischemia. These stress responses reduce the antioxidant capacity of cells and promote ROS production, leading to iron ion-induced mitochondrial damage and massive lipid peroxidation in neuronal cells, ultimately leading to ferroptosis [6]. Furthermore, Zhu K et al. showed that glycyrrhizin inhibited the onset of ferroptosis in neonatal rat HIBD neurons, inhibited oxidative stress, reduced mitochondrial damage and improved neuroinflammation via the HMGB1/GPX4 pathway [5]. This suggests that targeting ferroptosis in the treatment of HIBD is a promising avenue for us.

LCN2 is an inflammatory protein associated with different age-related CNS diseases and their risk factors [7]. Growing evidence that LCN2 regulates inflammatory cytokines and iron accumulation in the CNS reveals that LCN2 is a key mediator in the regulation of the CNS [8]. LCN2 controls cell cycle progression and death, self-renewal, proliferation and differentiation of neural stem cells and ultimately hippocampal function through the regulation of iron [9]. LCN2 has been shown to be dualistic in that it has a protective effect in acute CNS disorders but over time can have a detrimental effect on the nervous system [10]. Long-term damage to the cellular composition of hippocampal neurons by LCN2 ultimately leads to a decline in cognitive functions specific to spatial memory. However, it remains unknown whether LCN2 plays an equally critical role in HIBD. In addition, Zhou Y et al. noted that asiaticoside attenuates neonatal HIBD through inhibiting the TLR4/NF-κB/STAT3 pathway [11]. This implies to us that NF-κB/STAT3 is aberrantly activated in HIBD. More importantly, whether LCN2 is associated with NF-κB/STAT3 signaling in HIBD currently remains a mystery.

In this preliminary study, LCN2 was screened by bioinformatics as a key gene in ferroptosis in HIBD, and a novel diagnosis model for HIBD with remarkable performance was constructed based on six ferroptosis-HIBD genes. Subsequently, we investigated whether inhibition of LCN2 attenuated ferroptosis-related brain injury through in vitro experiments using LCN2 as a target. In addition, we further explored whether LCN2 may regulate glutamate-induced ferroptosis in HT22 cells through the NF-κB /STAT3 signaling pathway. The aim of this study was to reveal a novel function of LCN2 in ferroptosis in a model of HIBD and to identify a possible therapeutic biomarker that could provide a new target for HIBD treatment.

## 2. Materials and Methods

### 2.1. Data Acquisition and Processing

The ferroptosis-related genes were obtained from the Genecards website (http://www.genecards.org/ (accessed on 5 April 2022)) [12] using “ferroptosis” as a search keyword. The transcriptomic profiling (GSE23333), which was sequenced using microarray annotation platform GPL6885, and the gene expression matrix (GSE184997) related to HIBD were retrieved from the Gene Expression Omnibus (GEO) database (http://www.ncbi.nlm.nih.gov/geo/ (accessed on 4 April 2022)) for the treatment group and control group of the HI model in neonatal mice. The samples with the treatment of hypoxic pre-conditioning were excluded due to other possible causes of death. To ensure the accuracy of the results, GSE23333 (57 HIBD samples and 31 control samples) with a relatively large sample size was screened as a training dataset for subsequent analysis, and GSE184997 was screened as validation dataset. To improve data comparability, the “normalizeBetweenArrays” function in the “limma” software package of R [13] was used to normalize the datasets GSE184997 and GSE23333.

### 2.2. DEGs Screening and Gene Set Enrichment Analysis (GSEA)

Differential expression analysis was carried out after the selected gene expression matrix was normalized through the “limma” software package of R language [13]. The eligibility criteria of DEGs were |logFC| > 0.1, adjusted *p* value < 0.05. The data were visualized by the “ggplot2” package and “pheatmap” package in the R software. In order to investigate the potential mechanism between treatment group and control group of “mh.all.v2022.1.Mm.symbols.gmt”, which was downloaded from the molecular signature database (https://www.gsea-msigdb.org/gsea/index.jsp (accessed on 25 November 2022)) [14], the “clusterProfiler” R package [15] was used to respectively perform GSEA. The top three significantly enriched pathway gene sets were displayed. Statistical significance was set at a *p* value < 0.05.

### 2.3. Immune Infiltration Analysis through CIBERSORT

To explore the difference in immune cell infiltration between treatment group and control group, the CIBERSORT algorithm [16] was used to analyze the patterns of immune infiltration of HIBD. In order to accurately predict the composition of immune cells, the samples were screened according to the criterion of *p*-value < 0.05. Pearson’s correlation test and Student’s *t* test were performed to estimate the correlation between each type of immune cell and core ferroptosis-HIBD gene.

### 2.4. Weighted Gene Co-Expression Network Analysis (WGCNA)

In order to screen potential genes associated with HIBD, the expression matrix of DEGs was used to create a weighted gene co-expression network utilizing “WGCNA” R package [17]. Above all, a correlation matrix was obtained to assess the expression similarity of genes through clustering of all samples and calculating the Pearson correlation coefficient between each pair of genes. Next, the correlation matrix was converted into a weighted neighborhood matrix according to a suitable soft threshold, which is performed using the “pickSoftThreshold” function in WGCNA, and an optimal parameter β was selected to build a scale-free network. Then, the weighted adjacency matrix was transformed into a topological overlap matrix (TOM) [18] used as input for hierarchical clustering analysis [19]. To identify the significant modules, the correlation between module eigengenes (MEs) [20] and sample traits was assessed by Pearson’s correlation test. Therefore, the top two modules with high correlation coefficients were considered as HIBD-related modules.

### 2.5. Identification of Ferroptosis-HIBD Genes and Functional Enrichment Analysis

The genes in the HIBD-related modules of WGCNA and the ferroptosis-related genes obtained above were intersected to identify ferroptosis-HIBD genes for further analysis. The “clusterProfiler” package [15] in R was applied to perform gene ontology (GO) enrichment analyses [21] and Kyoto Encyclopedia of Genes and Genomes (KEGG) pathway analyses [22] based on ferroptosis-HIBD genes. Moreover, the biological processes (BP), cellular component (CC), and molecular function (MF) were investigated for GO enrichment analyses. A *p*-value < 1 was considered to be statistically significant.

### 2.6. ANN for Constructing an HIBD Classification Model

Six ferroptosis-HIBD genes were selected as disease-specific genes for subsequent model construction. The ferroptosis-HIBD genes’ expression data were converted to a gene score table based on the expression level. The gene score table was used to construct an ANN model through the R package “neuralnet” [23]. The model parameters were set to six input layers, five hidden layers, and two output layers. Additionally, we used the pROC package in R software (version 4.1.3) to calculate the areas under the receiver operating characteristic (ROC) curve, which were utilized for the robustness and clinical significance of the ANN model. The predictive performance of our model constructed in our study was then externally validated using GSE184997.

### 2.7. Construction of PPI Network and Identification of the Hub Genes

The PPI network was constructed using the online database of the Search Tool for the Retrieval of Interacting Genes (STRING; http://string-db.org/ (accessed on 18 July 2022)) [24] to estimate protein interactions of ferroptosis-HIBD genes. In addition, the network was visualized via the Cytoscape software (version 3.9.1) [25]. Concomitantly, the potential hub genes were detected based on the degree of nodes and the maximal clique centrality (MCC) algorithm through the CytoHubba [26] plugin of Cytoscape. Next, potential hub genes were screened with the “e1071” R package [27] using the SVM-RFE algorithm [28]. Moreover, the RF algorithm was performed to pick out potential hub genes by using the “randomForest” R package. Finally, the overlapped genes among potential hub genes generated by the PPI network, SVM-RFE algorithms and RF algorithm were regarded as ferroptosis-related hub genes of HIBD.

### 2.8. Validation of Expression Levels and the Diagnostic Value of the Core Hub Gene

The expression of ferroptosis-related hub genes of HIBD was analyzed by the “limma” package in R language. Logistic regression diagnostics were performed on LCN2 through a training dataset and validation dataset, and its confusion matrix was visualized to evaluate the prediction effect. An ROC curve was constructed, and area under the curve (AUC) value was used to distinguish the diagnostic validity of the HIBD treatment group and control group.

### 2.9. Cell Culture and Treatment

The HT22 cell line (a hippocampal neuronal cell line) was purchased from Procell Life Science & Technology Co., Ltd. (Wuhan, China). HT22 cells were cultured in Dulbecco’s modified Eagle’s medium (DMEM; Gibco, Grand Island, NE, USA) containing fetal bovine serum (FBS; Gibco, Grand Island, NE, USA) and 1% penicillin-streptomycin (PS; Gibco, Grand Island, NE, USA) and maintained in a humidified atmosphere of 95% air and 5% CO_2_ at 37 °C. HT22 cells were treated with glutamate (10 mM) for 24 h to induce ferroptosis. HT22 cells were inoculated in 6-well plates at a density of 3.5 × 10^5^ cells/well. After 24 h of treatment, they were divided into blank control, glutamate, and glutamate + Ferrostatin-1 (Fer-1) groups. HT22 cells were pretreated with Fer-1 (5 μM; Selleckchem, Houston, TX, USA) for 60 min prior to glutamate treatment.

### 2.10. Gene Knockdown by siRNA

HT22 cells were inoculated in 12-well plates at a density of 1.2 × 10^5^/well, and siRNA oligonucleotides targeting LCN2 (Sangon Biotech, Shanghai, China) were transfected in HT22 cells using the jetPRIME^®^ transfection reagent (Polyplus, France) for 36 h. The cells were then incubated with fresh DMEM replacement medium for 24 h. Si-NC was used as a negative control (Sangon Biotech, Shanghai, China). Various treatments were performed at 24 h after knockdown. To verify the validity of siRNA, Western blotting was used to detect the expression level of LCN2 protein.

The si-RNA nucleotide sequence was as follows:

si-NC, sense (5′-3′): UUC UCC GAA CGU GUC ACG UTT, antisense (5′-3′): ACG UGA CAC GUU CGG AGA ATT;
si-LCN2(1), sense (5′-3′): GGU CCA GAA AGA AAG ACA ATT, antisense (5′-3′): UUG UCU UUC UUU CUG GAC CTT;
si-LCN2(2), sense (5′-3′): GCU ACU GGA UCA GAA CAU UTT, antisense (5′-3′): AAU GUU CUG AUC CAG UAG CTT;
si-LCN2(3), sense (5′-3′): CCG ACC AAU GCA UUG ACA ATT, antisense (5′-3′): UUG UCA AUG CAU UGG UCG GTT.

### 2.11. Western Blotting

Cells were washed, collected and centrifuged at 4 °C for 5 min at 3000 rpm. Cells were lysed with radioimmunoprecipitation lysis solution (RIPA lysis solution; Solarbio, Beijing, China) containing phenylmethanesulfonyl fluoride (PMSF; Beyotime, Shanghai, China) and phosphatase inhibitors (Beyotime, Shanghai, China). Protein quantification was then performed using the BCA Protein Assay Kit (Yeasen, Shanghai, China). The proteins were then separated by 12% SDS-PAGE and transferred to a nitrocellulose membrane (NC). The membrane was closed for 2 h at room temperature in 5% (wt/vol) bovine serum albumin (BSA; Beyotime, Shanghai, China) and incubated overnight at 4 °C with primary antibody. After washing the antibody adequately, the membranes were incubated with horseradish peroxidase- (HRP-) linked secondary antibodies (1:4000 dilution; CST, Shanghai, China) for 1  h at room temperature. Finally, protein development was carried out using BeyoECL Moon (Beyotime Biotechnology, Shanghai, China). The primary antibodies used in this study were as follows: LCN2 (1:1000 dilution; ABclonal, Wuhan, China), ACSL4 (1:1000 dilution; ABclonal, Wuhan, China), 4-HNE (1:1000 dilution; Bioss Antibodies, Beijing, China), GPX4 (1:1000 dilution; CST, Shanghai, China), FTH1 (1:1000 dilution; CST, Shanghai, China), SLC7A11 (1:1000 dilution; CST, Shanghai, China), Mitofusin2 (1:1000 dilution; CST, Shanghai, China), VDAC (1:1000 dilution; CST, Shanghai, China), TOM20 (1:1000 dilution; CST, Shanghai, China), P-STAT3 (1:1000 dilution; ABclonal, Wuhan, China), STAT3 (1:1000 dilution; ABclonal, Wuhan, China), P65 (1:1000 dilution; CST, Shanghai, China), P-P65 (1:1000 dilution; CST, Shanghai, China), and GAPDH (1:1000 dilution; Sangon Biotech, Shanghai, China).

### 2.12. Lipid Peroxidation

Cells were seeded in 12-well plates at a density of 1.2 × 10^5^ or 1.8 × 10^5^ cells/well and then treated according to grouping. C11-BODIPY 581/591 (No. RM02821, ABclonal, Wuhan, China) was added at 10 μM per well and incubated at 37 °C for 1 h. Cells were then washed twice per well with 500 μL of phosphate buffer saline (PBS; Cytiva, Marlborough, MA, USA) to remove excess dye and resuspended in PBS containing 5% FBS. Finally, lipid peroxidation was detected in HT22 cells using a flow cytometer (DxP Athena 1L-3L, Cytek Biosciences, Fremont, CA, USA). Results were analyzed by FlowJo and GraphPad Prism (version 8) software.

### 2.13. Fe^2+^ Content

After treating the cells according to the grouping, the cells were washed two times using DMEM without FBS. Subsequently, Ferro Orange (1 μmol/L; λex: 543 nm, λem: 580 nm; Dojindo, Kumamoto, Japan) working solution was prepared using FBS-free DMEM according to the manufacturer’s instructions, incubated at 37 °C in a 5 % CO_2_ incubator for 30 min, and finally photographed by multifunctional microplate detection system (CYTATION5, BIOTEK, USA).

### 2.14. mtROS

Cells were incubated for 30 min at 37 °C protected from light using 2× mtROS™ 580 (λex: 500 nm, λem: 582 nm; AAT Bioquest, Pleasanton, CA, USA) working solution and washed three times with PBS according to the manufacturer’s instructions. Finally, the mtROS content in HT22 cells was determined using a flow cytometer (DxP Athena 1L-3L, Cytek Biosciences, USA). Results were analyzed by FlowJo and GraphPad Prism software.

### 2.15. Mitochondrial Membrane Potential (MMP)

Mitochondria store electrochemical potential energy in the inner mitochondrial membrane when they generate energy, and on both sides of the inner membrane, an asymmetric distribution of protons and other ion concentrations results in an MMP. Detection of MMP changes in HT22 cells using the JC-1 kit (Beyotime, Shanghai, China). The maximum excitation wavelength of JC-1 monomer is 514 nm, and the maximum emission wavelength is 529 nm; the maximum excitation wavelength of JC-1 polymer (J-aggregates) is 585 nm, and the maximum emission wavelength is 590 nm. Cells were treated according to the grouping requirements. Culture fluid was aspirated, cells were washed once with PBS, and 1 mL DMEM and 1 mL JC-1 staining working solution were added and mixed thoroughly. Cells were then incubated at 37 °C for 20 min in a cell incubator. At the end of incubation at 37 °C, the supernatant was aspirated and washed twice with JC-1 staining buffer (1×). Finally, 2 mL of DMEM was added, and imaging was performed using a multifunctional microplate detection system (CYTATION5, BIOTEK, USA).

### 2.16. Immunofluorescence (IF)

Cells were treated according to grouping requirements, washed three times using PBS, fixed with 4% paraformaldehyde (MACKLIN, Shanghai, China) for 20 min at room temperature, and permeabilized with 0.5% immunostaining permeabilization buffer with Triton X-100 (Beyotime, Shanghai, China) for 20 min. Thereafter, cells were blocked with 5% BSA for 30 min and incubated overnight at 4 °C with rabbit anti-P65 antibody (CST, Shanghai, China). Cells were then washed three times with PBS and labeled with the corresponding fluorescent rabbit secondary antibody (Invitrogen™, Shanghai, China) at a dilution of 1:800 for 1 h at room temperature. Nuclei were stained with 4′,6-diamidino-2-phenylindole (DAPI; CST, Shanghai, China) for 10 min. Final images were taken using a multifunctional microplate detection system (CYTATION5, BIOTEK, USA).

### 2.17. Transmission Electron Microscopy (TEM)

Observation of potential mitochondrial morphological changes in HT22 cells was performed using TEM. First, the cells were fixed with 2.5% glutaraldehyde in phosphate buffer for 4 h, followed by 1 % OsO4 in phosphate buffer for 2 h. Gradient ethanol was dehydrated, and acetone was used instead of ethanol. Cells were embedded in EPON 812 resin. Ultrathin sections 60 nm thick were cut and stained with uranyl acetate and lead citrate. Images were acquired under an HT7800 TEM (Hitachi, Tokyo, Japan).

### 2.18. Percentage of Cell Death

The percentage of cell death was detected using the phartmingen annexin V-FITC Apoptosis Detection Kit I (BD, USA), and the estimation procedure was performed according to the manufacturer’s instructions. Cells at a concentration of 4 × 10^5^ cells/well were seeded into 6-well plates. After attachment overnight, cells were pretreated with Fer-1 for 1 h before addition of glutamate. Twenty-four hours later, all cells, including those floating in the medium, were collected. Cells were resuspended in ice-water 1× binding buffer at a concentration of 1 × 10^6^ cells/mL. A 100 μL volume of cell suspension was mixed with 5 μL FITC Annexin V and 5 μL PI, respectively. The mixtures were incubated for 15 min at room temperature in the dark and then analyzed using a flow cytometer (DxP Athena 1L-3L, Cytek Biosciences, USA).

### 2.19. Cell Viability Assay

Cell viability was measured using the CCK-8 assay. HT22 cells were inoculated in 96-well plates at a concentration of 5 × 10^4^ cells/well. After incubation for 24 h, the cells were treated with different concentrations of glutamate for 12, 18 and 24 h. Then, 10 μL of CCK-8 solution was added directly to the culture medium (100 μL per well) and incubated at 37 °C for 3 h. The absorbance (Abs) of different groups was measured at 450 nm (n = 3). A blank group containing only culture medium and cells without any treatment was used as the control group. The cell survival rate was calculated as (Abs of experimental group − Abs of blank group)/(Abs of control group − Abs of blank group) × 100%.

### 2.20. Statistical Analysis

The correlations between WGCNA traits and gene expressions were detected using Pearson’s correlation test. Student’ s *t* test and Pearson’s correlation test were utilized to analyze the correlation between genes and immune cells. The PPI network was visualized via Cytoscape. Bioinformatics statistical analysis was performed using RStudio software (version 4.1.3). Statistical analyses were performed with GraphPad Prism version 8 (GraphPad Software, San Diego, CA, USA) and ImageJ software (version1.8.0.172). Differences between the two groups were tested by *t*-test and differences between the three groups were analyzed by one-way ANOVA. */# *p* < 0.05, **/## *p* < 0.01, ***/### *p* < 0.001, ****/#### *p* < 0.0001.

## 3. Results

### 3.1. DEG Analysis and GSEA

**Figure 1:**
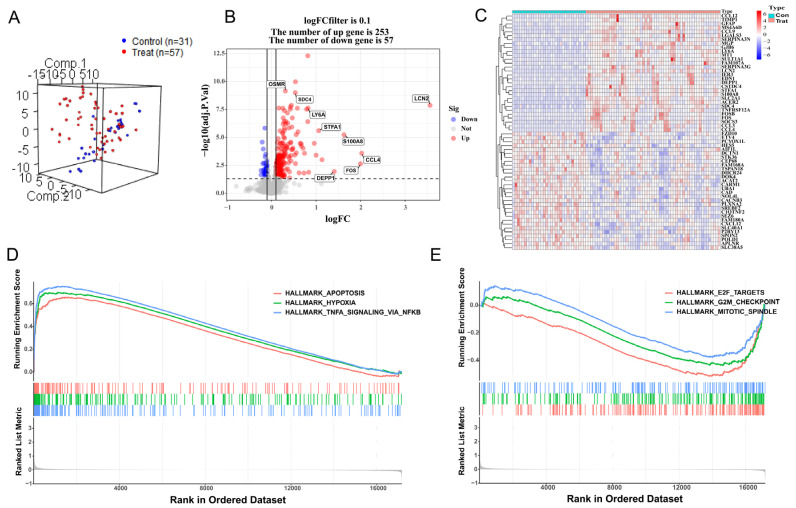
Differentially expressed gene analysis and gene set enrichment analysis. (**A**) Distribution of samples based on the whole genome expression data. (**B**) The volcano plot shows up-regulated and down-regulated genes. (**C**) Heatmap of the 60 most differentially expressed genes based on GSE23333. Light blue is the control group, red is the treatment group, and the color intensity (from red to blue) indicates the higher to lower expression. (**D**) Three up-regulated pathways. (**E**) Three down-regulated pathways.

After preprocessing the training datasets and eliminating outliers, the mouse samples were reduced to 57 HIBD samples and 31 control samples, and 17,177 gene expression profiles were obtained from 88 samples. Normalization of these two gene expression profiles was conducted through the limma software package in R language (GSE23333: Supplementary Figure S1A,B; GSE184997: Supplementary Figure S1D,E). The control group and treatment group were decentralized according to the normalized gene dataset (Figure 1A). A total of 310 DEGs were screened by setting the cutoff value to adjusted *p* value < 0.05 and ∣logFC ∣> 0.1 as the criteria, of which 253 were up-regulated genes and 57 were down-regulated genes. A density plot demonstrated the distribution of logFC base on up-regulated and down-regulated genes (Supplementary Figure S1C). The differences between up-regulated genes and down-regulated genes were presented in a volcano plot (Figure 1B), and a heatmap showed the bivariate hierarchical clustering results of the top 60 DEGs (Figure 1C). To elucidate underlying regulatory mechanisms in HIBD, a GSEA analysis was performed. The gene sets presented high expression in pathways including HALLMARK_ APOPTOSI, HALLMARK_ HYPOXIA, and HALLMARK_TNFA_SIGNALING_VIA_NFKB (Figure 1D; all *p* < 0.05). However, the GSEA results showed that gene sets presented low expression in HALLMARK_MITOTIC_SPINDLE, HALLMARK_G2M_CHECKPOINT, and HALLMARK_E2F_TARGETS (Figure 1E; all *p* < 0.05).

### 3.2. Construction of Weighted Co-Expression Network and Screening of Significant Modules

**Figure 2:**
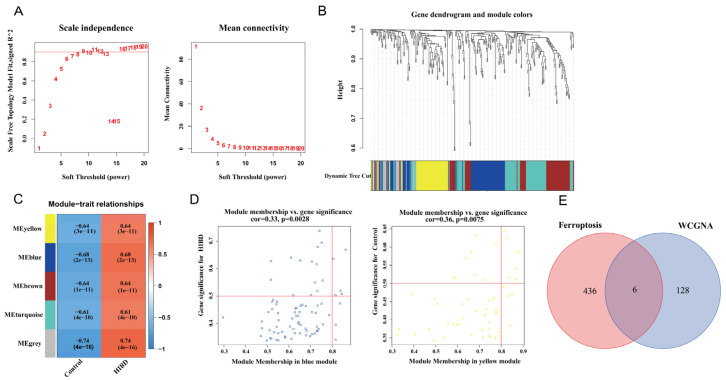
Weighted co-expression network analysis. (**A**) Assessment of scale-free fit index (left) and mean connectivity (right) for distinct soft threshold powers. (**B**) Dendrogram of clustering of differentially expressed genes based on topological overlap matrix. (**C**) Heatmap of the correlation between modules and sample traits. (**D**) Two scatter plots of gene significance for HIBD vs. module membership in the blue module and yellow module. (**E**) Venn plot for intersected genes in WGCNA modules and ferroptosis-related genes.

Before constructing the weighted co-expression network, β = 9 was chosen as the optimum soft-thresholding power value to ensure a scale-free network when the scale-free R^2^ reached 0.9 (Figure 2A). Following the determination of the soft threshold, the adjacency matrix was calculated and subsequently converted into TOM. Based on TOM, a dendrogram was constructed using hierarchical clustering, and a total of five modules were then generated (Figure 2B). After that, the modules that highly correlated with HIBD were picked out based on the correlations between MEs, as shown in Figure 2C. The heatmap of the module–trait relationship showed that MEyellow and MEblue had a strong correlation with HIBD (Figure 2C). High correlation coefficients of gene significance and module membership were revealed in the blue module (cor = 0.33, *p* = 0.0028) and yellow module (cor = 0.36, *p* = 0.0075) (Figure 2D). Via intersecting the genes in the HIBD-related modules of WGCNA and ferroptosis-related genes, six overlapping genes were obtained (Figure 2E).

### 3.3. GO and KEGG Enrichment Analysis

The results of KEGG pathway enrichment analysis illustrated that the ferroptosis-HIBD genes may influence the occurrence and progression of HIBD by being involved in ferroptosis, arginine and proline metabolism, IL-17 signaling pathway, etc. (Supplementary Figure S2A,B). GO analysis showed that in CC, ferroptosis-HIBD genes were associated with ficolin−1−rich granule membrane, tertiary granule, ficolin−1−rich granule, and secretory granule lumen (Supplementary Figure S2C). In BP, ferroptosis-HIBD genes were mainly enriched in the positive regulation of ion transport, regulation of metal ion transport, calcium ion transport, and response to increased oxygen levels (Supplementary Figure S2D). In MF, ferroptosis-HIBD genes were mainly involved in extracellular matrix binding, protease binding, and iron ion binding (Supplementary Figure S2E).

### 3.4. Establishment and Validation of ANN Model

**Figure 3:**
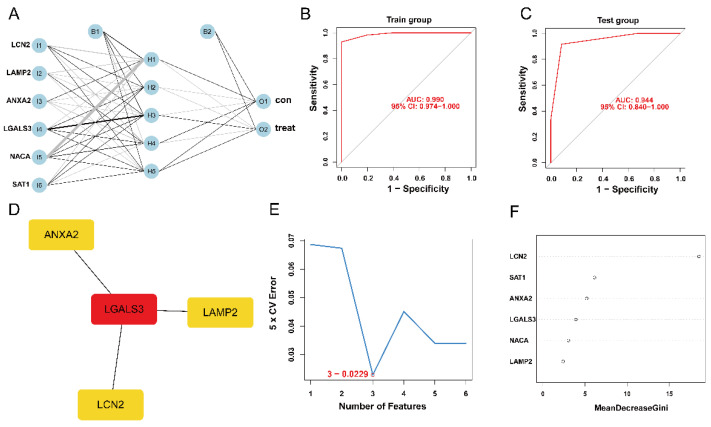
The construction of HIBD classification model and identification of gene biomarkers for HIBD. (**A**) Visualization of artificial neural network results. (**B**,**C**) The ROC curves were utilized to evaluate the performance of the HIBD classification model in training and validation datasets. (**D**) CytoHubba detected four ferroptosis-HIBD genes. (**E**) Three genes were identified by the SVM-RFE algorithm with an error of 0.0229. (**F**) A minimum error regression tree was established for six ferroptosis-HIBD genes.

The expression status levels of the six ferroptosis-HIBD genes differed significantly between HIBD and normal control based on GSE23333 data (Supplementary Figure S3A–F). Subsequently, an ANN model for an HIBD classification model based on the six ferroptosis-HIBD genes was established, with six input layers, five hidden layers, and two output layers (Figure 3A). Based on calculations with the pROC package, the model’s prediction accuracy was 0.990 in GSE23333 (Figure 3B) and 0.944 in GSE184997 (Figure 3C), which indicated that our model classification performance was robust and stable.

### 3.5. Construction of PPI Network and Identification of the Hub Genes

**Figure 4:**
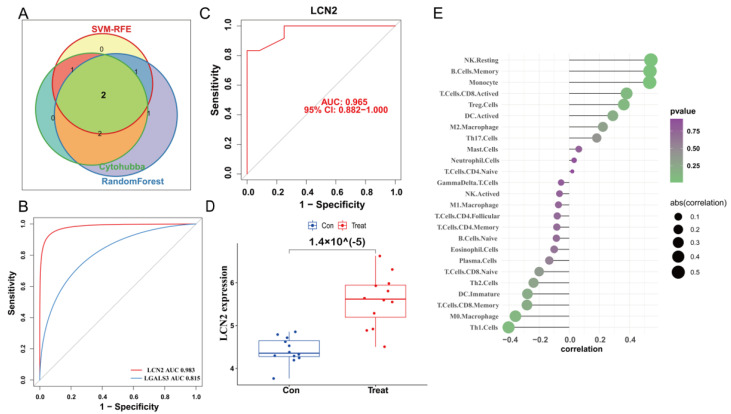
Validation of the diagnostic value of the hub gene. (**A**) Venn diagram indicates the overlapping genes in SVM-RFE algorithm, random forest algorithm and CytoHubba. (**B**) The comparison of two potential biomarkers via GSE23333. (**C**) ROC curves of LCN2 in dataset GSE184997. (**D**) The expression of LCN2 in the dataset GSE184997. (**E**) Lollipop plot showing the correlation of immune cell infiltration and LCN2.

We used the STRING database to construct a PPI network for the ferroptosis-HIBD genes and visualized them using Cytoscape software with the purpose of investigating the protein interactions of the target genes. The top four genes with a high number of adjacent nodes were screened as hub genes LCN2, LGALS3, LAMP2, and ANXA2 (Figure 3D). To further identify gene biomarkers of HIBD from ferroptosis-HIBD genes, a machine learning algorithm was selected and implemented. The SVM-RFE algorithm revealed that the SVM-RFE model based on three hub genes showed the best error rate (0.0229, Figure 3E) and the best accuracy rate (0.977, Supplementary Figure S4C). Therefore, LGALS3, LCN2, and NACA were identified as potential hub genes. Meanwhile, the RF algorithm identified the top six genes, including LCN2, ANXA2, SAT1, NACA, LAMP2, and LGALS3 (Figure 3F). Based on the correlation plot between the number of RF trees and model error (Supplementary Figure S4B), 37 trees were chosen as the final model’s parameter. Finally, the overlapping genes among potential hub genes generated by CytoHubba, SVM-RFE algorithm and RF algorithm were considered as ferroptosis-related core genes in HIBD (Figure 4A).

### 3.6. Validation of LCN2 and Correlation Analysis between LCN2 and Immune Cell Infiltration

The ROC curve of the training dataset showed that the AUC of LCN2 was 0.983, which was higher than that of LGALS3 (AUC = 0.815) (Figure 4B). Meanwhile, LCN2 was verified via validation dataset GSE184997, and the AUC was 0.965 (Figure 4C). The confusion matrix was visualized to assess the predictive effect of LCN2 in the training and validation datasets, and the results showed that LCN2 had accurate predictive properties (Supplementary Figure S4D,E). In the validation dataset, the *p* value revealed the expressional differences in LCN2 between control and treat groups, and the expression levels of LCN2 in the control group were significantly lower than those in the treat group (*p* < 0.05) (Figure 4D). Therefore, LCN2 was chosen as the core hub gene of HIBD following a comprehensive analysis of all of these parameters. The result of correlation analysis indicated that LCN2 was positively correlated with Monocyte (r = 0.53, *p* = 0.01), NK Resting (r = 0.54, *p* = 9.04 × 10^−3^), and B Cell Memory (r = 0.54, *p* = 1.00 × 10^−2^) (Supplementary Figure S4E–H).

### 3.7. LCN2 Is Highly Expressed in Glutamate-Induced HT22 Cell Model, Which Can Be Reversed by Ferroptosis Inhibition

**Figure 5:**
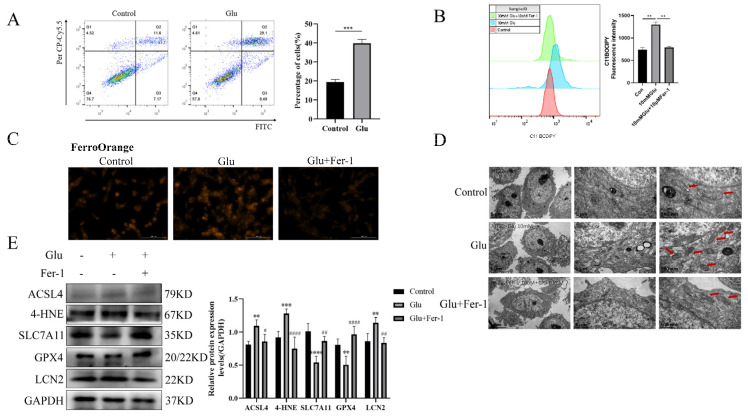
Glutamate stimulation of HT22 cells induces high expression of LCN2 in ferroptosis. Glutamate stimulation of HT22 cells with or without pre-treatment with Fer-1. (**A**) Representative results of annexin V-FITC/PI staining and quantitative analysis after the treatment (glutamate 10 mM) for 24 h. The mean ± SD is shown. Unpaired two-tailed t test was used for statistical analysis. *** *p* < 0.001. (**B**) Detection of C11-BODIPY 581/591 fluorescence of HT22 cells by flow cytometry. Glutamate: 10 mM, Fer-1: 10 μM, treatment time: 24 h. **: It represents the glutamate stimulation group compared with the control group or glutamate combined with Fer-1 group, *p* < 0.01. (**C**) Detection of Ferro Orange fluorescence of HT22 by multifunctional microplate detection system. Glutamate: 10 mM, Fer-1: 5 μM, treatment time: 24 h. Scale bar is 100 μm. (**D**) Representative TEM images of the untreated group, the glutamate-treated group and the glutamate-co-administered Fer-1 group. The red arrows represent mitochondria. Glutamate: 10 mM, Fer-1: 10 μM, treatment time: 24 h. Scale bar = 500 nm, 1 μm, 5 μm. (**E**) Western blotting analysis and quantitative analysis of ACSL4, 4HNE, SLC7A11, LCN2, GPX4 and GAPDH expression in HT22. Glutamate: 10 mM, Fer-1: 5 μM, treatment time: 24 h. **/***/****: It indicates that the glutamate-treated group compared to the blank control group, ** *p* < 0.01, *** *p* < 0.001, **** *p* < 0.0001. #/##/####: It indicates that pretreatment of the glutamate group with Fer-1 compared to that without Fer-1, # *p* < 0.05, ## *p* < 0.01, #### *p* < 0.0001. Two-way ANOVA multiple comparisons were performed between groups.

To validate the possibility of bioinformatic screening of LCN2 as a biomarker for the ferroptosis-associated gene HIBD, glutamate-stimulated HT22 cells were used for 24 h. First of all, we examined the changes in HT22 cell viability when exposed to different concentrations of glutamate by the CCK-8 assay. The experimental results showed that glutamate toxicity to HT22 cells increased with increasing time and concentration (Supplementary Figure S5). Moreover, we performed an annexin-V/propidium iodide (AV/PI) dual staining assay followed by flow cytometry. The experimental results showed that a significant increase in the number of dead cells was observed in HT22 cells when exposed to glutamate (Figure 5A). Lipid peroxidation was detected by flow cytometry and was significantly elevated in glutamate-stimulated HT22 cells (Figure 5B). To further determine the presence of ferroptosis in glutamate-stimulated HT22 cells, we examined the mitochondrial structure and Fe^2+^ content. Notably, glutamate stimulation of HT22 cells significantly altered mitochondrial structure, with a reduction in mitochondrial cristae and an increase in membrane density, as well as an increase in Fe^2+^ content (Figure 5C,D). Ferro Orange is a fluorescent probe that specifically detects unstable iron (II) ions (Fe^2+^) and is localized to the endoplasmic reticulum. Consistent with the bioinformatic screening results, LCN2 was highly expressed in the glutamate-stimulated HT22 group (Figure 5E). In addition, the results of Western blotting experiments showed that the expression levels of ACSL4 and 4HNE proteins were increased in glutamate-stimulated HT22 cells, while the expression levels of SLC7A11 and GPX4 proteins were decreased (Figure 5E). This further suggests that glutamate induces ferroptosis in HT22 cells. The use of Fer-1 (a ferroptosis inhibitor; the anti-ferroptotic activity of Fer-1 is actually due to the scavenging of initiating alkoxyl radicals produced, together with other rearrangement products, by ferrous iron from lipid hydroperoxides [29]) reversed all of these trends (Figure 5A–E). In conclusion, LCN2 plays an important role in the regulation of glutamate-stimulated ferroptosis in HT22 cells.

### 3.8. Glutamate Induced Mitochondrial Dysfunction in HT22 Cells

**Figure 6:**
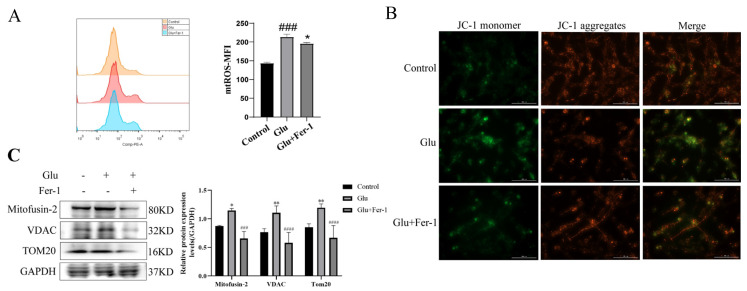
Glutamate stimulation of HT22 cells causes mitochondrial damage. For all experiments, glutamate: 10 mM, Fer-1: 5 μM, treatment time: 24 h. (**A**) HT22 cells were treated with indicated drugs for 24 h, and the level of mtROS was detected by flow cytometry with mtROSTM 580 (n = 3). ### *p* < 0.001 compared with the normal group; glutamate combined with Fer-1 group compared with glutamate group, * *p* < 0.05. (**B**) JC-1 kit was used to evaluate the effect of glutamate stimulation on MMP in HT22 cells. Red fluorescence: aggregates; green fluorescence: monomer; yellow fluorescence: combined. Scale bar is 100 μm. (**C**) The expression levels of Mitofusin2, VDAC and TOM20 in different groups were detected by Western blotting, and to quantify and analyze. */**: It indicates that the glutamate-treated group compared to the blank control group, * *p* < 0.05, ** *p* < 0.01. ###/####: It indicates that pretreatment of the glutamate group with Fer-1 compared to that without Fer-1, ### *p* < 0.001, #### *p* < 0.0001. Two-way ANOVA multiple comparisons were performed between groups.

Since HIBD, ferroptosis and ROS production are closely related, and mitochondria are important organelles for ROS production, we hypothesized that LCN2 regulation of glutamate-induced ferroptosis in HT22 cells is associated with mitochondrial dysfunction. We determined the effects of glutamate-stimulated HT22 cells on mtROS and MMP using mtROSTM 580 staining and JC-1 staining, respectively. Flow cytometry results showed that glutamate induction of HT22 cells significantly increased mtROS production (Figure 6A). The effect of glutamate stimulation of HT22 cells on MMP was observed using a multifunctional microplate detection system. When the mitochondrial membrane potential is high, JC-1 aggregates in the mitochondrial matrix (matrix) form polymers (J-aggregates), which can produce red fluorescence; when the mitochondrial membrane potential is low, JC-1 cannot aggregate in the mitochondrial matrix when JC-1 is a monomer, which can produce green fluorescence. The fluorescence of JC-1 aggregates was significantly decreased in glutamate-stimulated HT22 cells compared to controls, indicating a decrease in MMP (Figure 6B). More importantly, the trend of mtROS and MMP could be reversed using Fer-1 (Figure 6A,B). This implies that ferroptosis in our glutamate-stimulated HT22 cells may be mediated by mitochondrial dysfunction. To further clarify the association, the protein expression levels of Mitofusin2, VDAC, and TOM20 were examined by Western blotting assays. Glutamate stimulation of HT22 cells significantly increased the expression levels of Mitofusin2, VDAC and TOM20 compared to the control group, while the addition of Fer-1 down-regulated their expression levels (Figure 6C). This revealed that glutamate stimulation of HT22 cells significantly disrupted the structural integrity of mitochondria and produced dysfunction, which was rescued by the use of Fer-1. All of the above data suggest that glutamate induces mitochondrial dysfunction-mediated ferroptosis in HT22 cells.

### 3.9. Knockdown of LCN2 Rescues Glutamate-Induced Mitochondria-Mediated Ferroptosis in HT22 Cells

**Figure 7:**
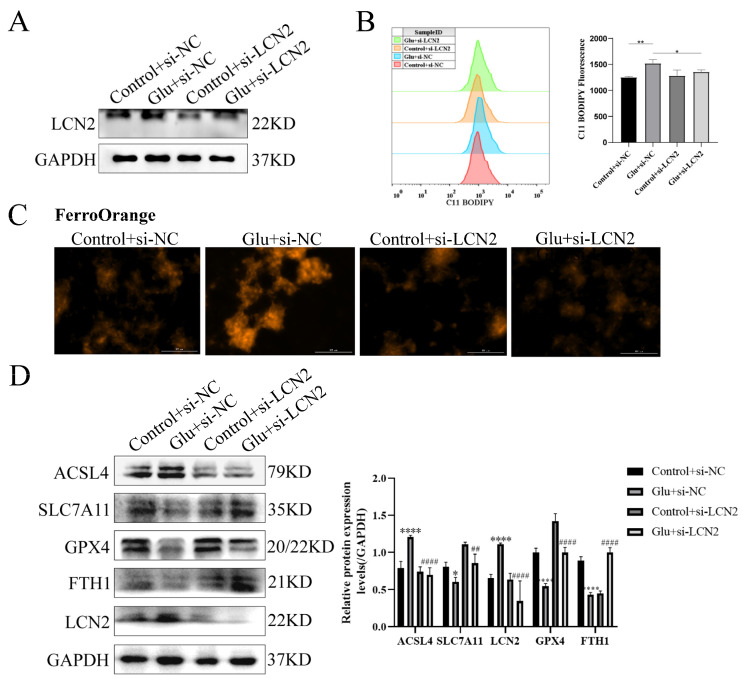
LCN2 silencing inhibits glutamate-induced ferroptosis in HT22 cells. Using specific siRNA knockdown of LCN2, process accordingly according to grouping requirements. Glutamate: 10 mM. (**A**) Knockdown of LCN2 by specific si-RNAs in HT22, after 72 h, and the expression of LCN2 and GAPDH were detected by Western blotting. (**B**) After specific knockdown of LCN2 for 36 h, HT22 cells were treated with glutamate for 24 h, followed by flow cytometry to detect lipid peroxidation. *: *p* < 0.05. **: *p* < 0.01. (**C**) Representative images showing the amount of Ferro Orange in the indicated cells. Scale bar is 100 μm. (**D**) Levels of ACSL4, SLC7A11, FTH1, GPX4, LCN2 and GAPDH proteins in the indicated cells were assessed by Western blotting. */****: It indicates that the glutamate-treated group compared to the blank control group, * *p* < 0.05, **** *p* < 0.0001. ##/####: It indicates that pretreatment of the glutamate group with Fer-1 compared to that without Fer-1, ## *p* < 0.01, #### *p* < 0.0001. Two-way ANOVA multiple comparisons were performed between groups.

To determine whether LCN2 could protect against neuronal injury after HIBD by inhibiting ferroptosis, we next assessed the effect of LCN2 knockdown on glutamate-stimulated ferroptosis in HT22 cells. First, we examined LCN2 knockdown efficiency by Western blotting assay, which showed a good second knockdown (Figure 7A), so we next chose to use a second si-LCN2 for subsequent experiments. Flow cytometry assays of C11 BODIPY 581/591 fluorescent probe staining showed that LCN2 knockdown significantly inhibited the elevated glutamate-stimulated lipid peroxidation in HT22 (Figure 7B). Importantly, we found by looking at Ferro Orange fluorescent probe staining that LCN2 knockdown significantly reduced Fe^2+^ content and inhibited iron accumulation compared to glutamate-stimulated HT22 cells (Figure 7C). ACSL4 promotes lipid peroxidation in polyunsaturated fatty acids (PUFAs) [30]; SLC7A11, GPX4 and FTH1 were consumed at the time of the ferroptosis event [31]. In addition, the LCN2 knockdown group showed significantly lower levels of the ferroptosis-related gene ACSL4 protein and higher levels of SLC7A11, GPX4 and FTH1 protein expression compared to the glutamate-stimulated HT22 cell group (Figure 7D). These results suggest that LCN2 gene deficiency inhibits the occurrence of neuronal ferroptosis in glutamate-stimulated HT22.

### 3.10. LCN2 Inhibits Glutamate-Induced Mitochondria-Mediated Ferroptosis in HT22 Cells through NF-κB/STAT3 Axis

**Figure 8:**
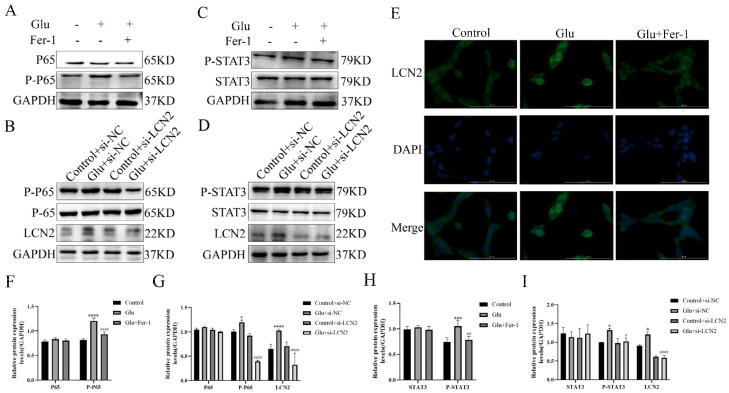
LCN2 regulates the activation of the NF-κB /STAT3 axis in glutamate-stimulated HT22 cells. (**A**–**D**,**F**–**I**) Western blotting was used to detect the protein expression of P-P65, P65, STAT3, P-STAT3, LCN2 and GAPDH in different treated HT22 cells. Quantitative analysis of results. Compared with the addition of the si-NC empty carrier group, * *p* < 0.05, *** *p* < 0.001, **** *p* < 0.0001. Compared to the group treated with glutamate stimulation combined with si-NC, # *p* < 0.05, ## *p* < 0.01, #### *p* < 0.0001. Two-way ANOVA multiple comparisons were performed between groups. (**E**) Immunofluorescence images of HT22 cells labeled with P65 antibody. Co-staining with DAPI (blue) to show the nucleus. Scale bar = 100 μm. For all experiments, glutamate: 10 mM, Fer-1: 5 μM.

To test whether the NF-κB pathway is involved in LCN2 regulation of mitochondria-mediated ferroptosis in glutamate-stimulated HT22, we examined the expression of NF-κB pathway-related proteins. Glutamate stimulation of HT22 cells significantly increased the protein expression content of phosphorylated P65 (P-P65) compared to the untreated group, whereas the use of Fer-1 restored it to normal levels (Figure 8A,F). To further confirm that LCN2 regulates ferroptosis in glutamate-stimulated HT22 cells through activation of the NF-κB signaling pathway, we observed the localization of P65 by cellular IF assay, and the results showed that glutamate treatment promoted nuclear translocation of P65, indicating that the NF-κB pathway was activated in glutamate-stimulated HT22 cells (Figure 8E). In addition, Western blotting experiments showed that LCN2 knockdown significantly reduced the protein expression of P-P65 (Figure 8B,G), suggesting that LCN2 knockdown inhibited activation of the NF-κB signaling pathway in glutamate-stimulated HT22 cells.

As STAT3 is closely associated with mitochondrial function, we sought to explore whether STAT3 is involved in LCN2 regulation of mitochondrial dysfunction in glutamate-stimulated HT22 cells. Phosphorylated STAT3 (P-STAT3) protein expression was elevated in glutamate-stimulated HT22 cells compared to the untreated group, and the addition of Fer-1 restored its levels (Figure 8C,H). In addition, silencing LCN2 expression using si-LCN2 reduced P-STAT3 expression (Figure 8D,I), and these data suggest that LCN2 promotes glutamate-stimulated mitochondria-mediated ferroptosis in HT22 cells through activation of STAT3. LCN2 promotes glutamate-induced mitochondria-mediated ferroptosis in HT22 cells through activation of NF-κB/STAT3.

## 4. Discussion

Several studies have shown that ferroptosis is critical to the onset and progression of neurodegenerative diseases and that targeting ferroptosis may be a promising therapeutic approach for neonatal HIBD. However, there are fewer studies on the role of ferroptosis in HIBD. Therefore, the identification of key diagnostic and prognostic biomarkers for HIBD remains critical. Advances in machine learning and public gene expression data have made it feasible to infer biomarkers for disease diagnosis and prognosis. The benefits of RF include relatively good accuracy, precision and ease of use to help identify key genes [32]. The highlight of our study is the innovative use of ANN methods with good predictive performance and the application of PPI networks and integration of two machine learning algorithms to further identify core diagnostic biomarkers. Several diseases, including Alzheimer’s disease, acute myocardial infarction and periodontitis, have already benefited from the ANN approach [33,34,35]. In this study, we first screened LCN2 as a key gene for ferroptosis in HIBD by bioinformatic means. We then constructed a glutamate-stimulated HT22 cell induction model to demonstrate in vitro that LCN2 was highly expressed in HIBD and that LCN2 silencing inhibited glutamate-stimulated ferroptosis in HT22 cells. We also found that glutamate stimulation of HT22 cells induced mitochondrial dysfunction. Furthermore, NF-κB and STAT3 were activated in the glutamate-stimulated HT22 cell model, and silencing LCN2 inhibited their activation. Thus, we conclude that LCN2 regulates mitochondria-mediated ferroptosis in HIBD through activation of the NF-κB /STAT3 axis.

LCN2 is an acute-phase protein that, by binding to iron-loaded siderophores, acts as a potent bacteriostatic agent in the iron-depletion strategy of the immune system to control pathogens [36]. The balance of iron homeostasis plays an important role in maintaining normal CNS function, and there is growing evidence that LCN2 plays a key role in maintaining iron homeostasis in the CNS. Either iron reduction or iron overload can trigger neurodegenerative diseases. LCN2 has now emerged as a potential clinical biomarker for multiple sclerosis [37], ageing-related cognitive decline [38] and neuropsychiatric lupus [39]. Studies have shown that blood levels of LCN2 protein increase with age and mild cognitive impairment in different diseases of the CNS, including Alzheimer’s disease, Parkinson’s disease and multiple sclerosis, and that brain tissue levels of LCN2 protein increase in people after death [37,40,41,42,43]. LCN2, a regulator of ferroptosis, now plays an important role in a variety of diseases—for example, in liver cancer [44], colorectal cancer [45], etc. However, it is not clear whether LCN2 plays an important role in the pathogenesis of ferroptosis in HIBD. Our study points to LCN2 as a potential ferroptosis-related biomarker in HIBD, and knockdown of LCN2 can inhibit the occurrence of ferroptosis in HIBD and rescue neuronal death.

Ferroptosis is a novel form of cell death caused by the massive accumulation of lipid hydroperoxides catalyzed by free iron, which ultimately leads to cell death [4]. Ferroptosis involves three main factors: increased intracellular free iron, depletion of glutathione/GPX4/system xc- and peroxidation of membrane PUFAs [46,47,48]. A growing body of data suggests that the pathophysiological changes of oxidative apoptosis and ferroptosis are also observed in a variety of neurodegenerative diseases as well as in the aging brain. Our study shows that ferroptosis is involved in glutamate-induced neurotoxicity in HT22 cells. Inhibition of post-hypoxic-ischemic neuronal ferroptosis is an important strategy in the treatment of HIBD. Zhu K et al. showed that TLR4 was highly expressed in HIBD and activated ferroptosis. In contrast, TLR4 inhibition attenuated oxidative stress-induced injury, reduced the activation of ferroptosis and attenuated neuroinflammation in HIBD subjects [49]. In addition, Cai Y et al. also found that Vitamin D inhibited HIBD ferroptosis and improved mitochondrial and oxidative damage through activation of the NRF2/HO-1 pathway [50]. However, potentially relevant biomarkers of ferroptosis in the regulation of ferroptosis in HIBD remain to be discovered. Our study indicates that LCN2, a key regulator of ferroptosis, plays an integral role in the regulation of ferroptosis in HIBD.

The NF-κB pathway is the main signaling pathway that activates LCN2 transcription. Secondary brain injury resulting from neuronal ferroptosis and over-activation of the inflammatory response plays an important role in hypoxic ischemic brain injury [51]. Although previous studies have shown that NF-κB is involved in the neuroinflammatory response induced by brain injury, the role and mechanism of NF-κB-mediated transcriptional activation of LCN2 in neonatal HIBD is unclear. For example, Huang Z et al. showed that vitamin D promotes the cisplatin sensitivity of oral squamous cell carcinoma by inhibiting LCN2-modulated NF-κB pathway activation through RPS3 [52]. Therefore, we also tested whether the NF-κB pathway was activated in glutamate-stimulated ferroptosis in HT22 cells. The results showed that glutamate-stimulated P-P65 expression was increased in HT22 cells, promoting P65 nuclear translocation and activation of the NF-κB signaling pathway, whereas inhibition of LCN2 did not activate the NF-κB pathway.

However, there are several limitations to our study. First, we only used one dataset to build the model, which is not a large data sample for machine learning. More research data will be needed in the future to test the reliability of the model. Secondly, as different species were used for the study, detailed information was lacking, and some important clinical characteristics such as age and gender were not integrated into the diagnostic model. Third, we only showed a link between LCN2 and neuronal ferroptosis; other modes of neuronal death, including apoptosis, autophagy and pyroptosis, were not examined here. Fourth, we have not validated this in an animal model of HIBD and lack some in vivo experimental evidence. Fifth, our study confirmed that glutamate-stimulated HT22 cells produce mitochondrial dysfunction and activate STAT3. However, this study did not further explore whether LCN2 silencing reverses its outcome. Therefore, these limitations need to be further addressed in subsequent studies.

## 5. Conclusions

Taken together, our findings confirmed that the expression level of LCN2 was significantly associated with the risk of HIBD and developed a new model for the classification of HIBD. More importantly, we found for the first time that LCN2 promotes the development of ferroptosis in HIBD neurons through the NF-κB /STAT3 pathway and induces mitochondrial dysfunction. In conclusion, these findings highlight the potential of LCN2 as a key factor for ferroptosis in the treatment of HIBD, provide evidence for the identification of LCN2 as a biomarker for HIBD, and offer new targets and therapeutic strategies for diseases characterized by ferroptosis, especially HIBD.

## Data Availability

The data used to support the findings of this study are included within the article.

## Supplementary Materials

The following supporting information can be downloaded at: https://www.mdpi.com/article/10.3390/antiox12010186/s1, Figure S1: Data preprocessing; Figure S2: Functional enrichment analysis of ferroptosis-HIBD genes; Figure S3: The expression of Ferroptosis-HIBD genes; Figure S4: Identification of two gene biomarkers for HIBD and correlation analysis of core gene and three immune cells; Figure S5: The effect of glutamate treatment on HT22 cells.
