# Supplementary material for: Identification of Lipocalin 2 as a Ferroptosis-Related Key Gene Associated with Hypoxic-Ischemic Brain Damage via STAT3/NF-κB Signaling Pathway
Source: Antioxidants (Basel). 2023 Jan 12;12(1):186. doi: 10.3390/antiox12010186 (PMC9854551; doi:10.3390/antiox12010186)
Supplement: Supplementary file 1 [file antioxidants-12-00186-s001.zip › antioxidants-2099852-supplementary.pdf]

Supplementary materials:

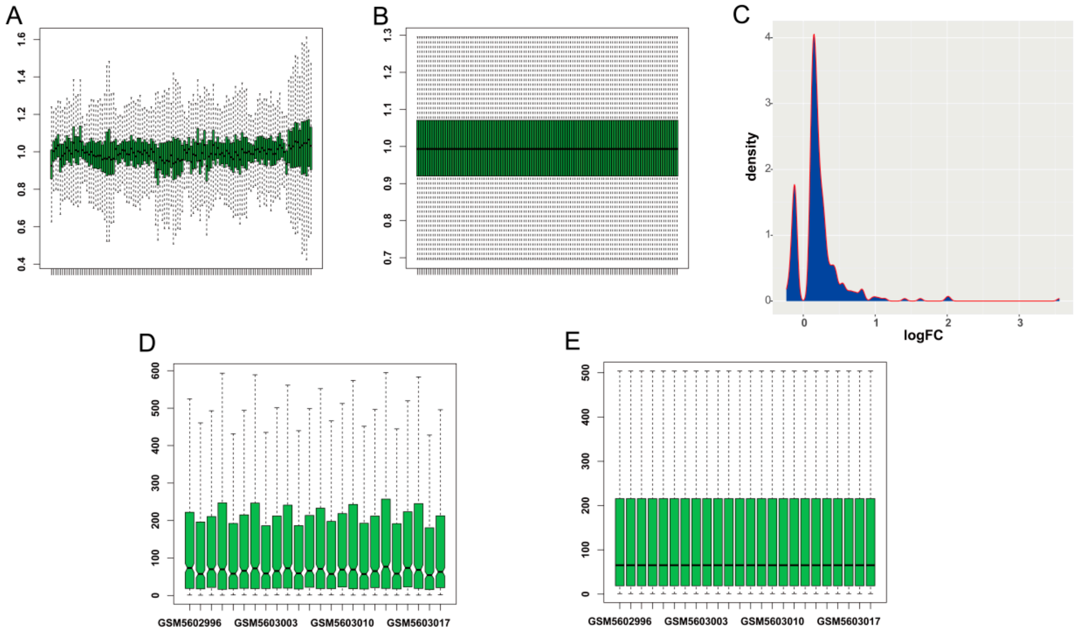

Figure S1. Data preprocessing. (A-B) Boxplots were performed to show data before (A) and after (B) normalizing the data GSE23333. (C)Density diagram showed logFC of DEGs. (D-E) Boxplots were performed to show data before (D) and after (E) normalizing the data GSE184997.

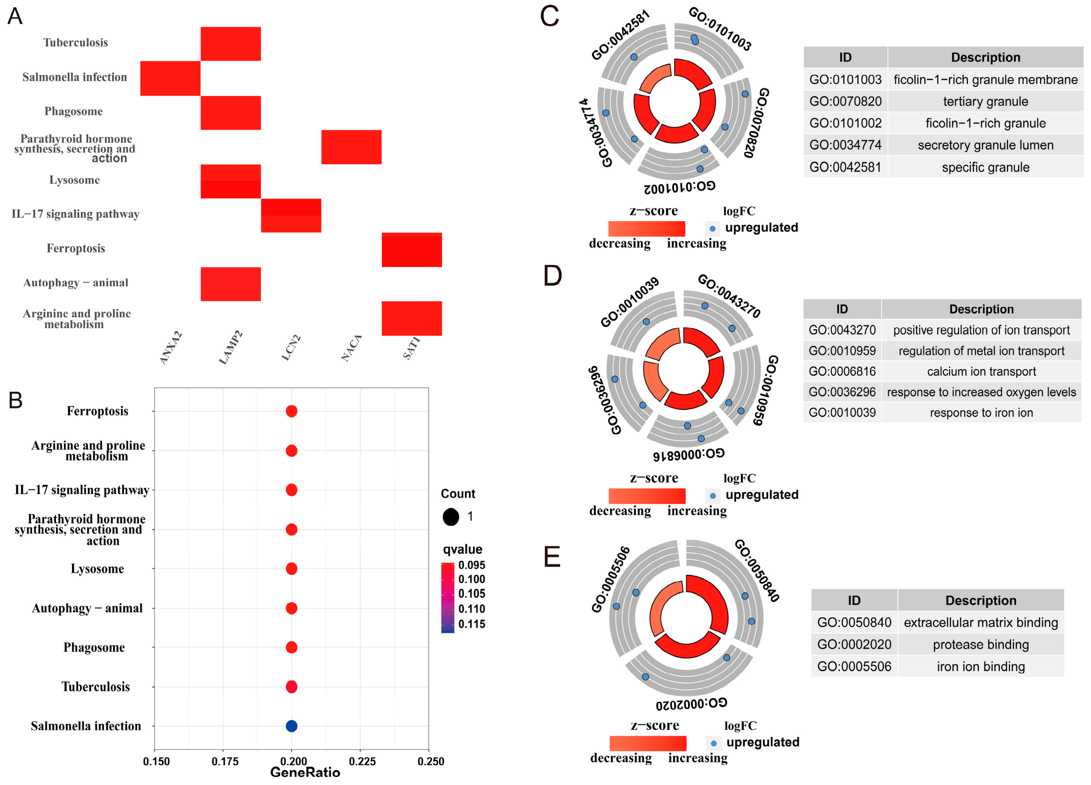

Figure S2. Functional enrichment analysis of ferroptosis-HIBD genes. (A)The pathway enriched by each ferroptosis-HIBD genes. (B) Kyoto Encyclopedia of Genes and Genomes enrichment analysis. (C-E) Gene

Ontology enrichment analysis, including cell composition, biological process, and molecular function.

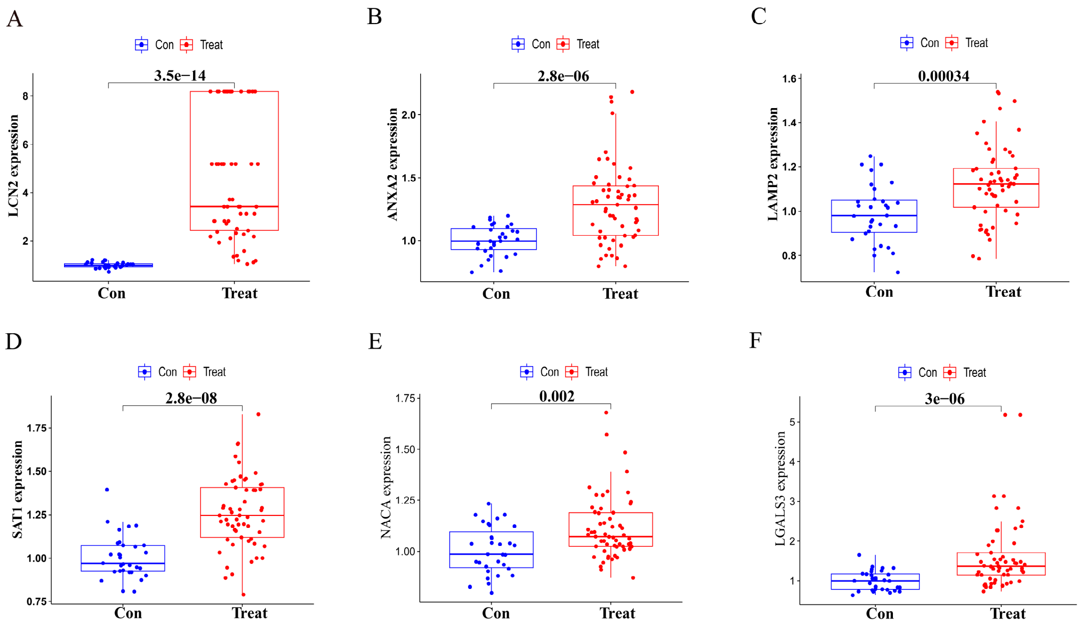

Figure S3. The expression of Ferroptosis-HIBD genes. (A-F) The expression of ferroptosis-HIBD genes in the dataset GSE23333. *P <0.05, **P <0.01, ***P <0.001.

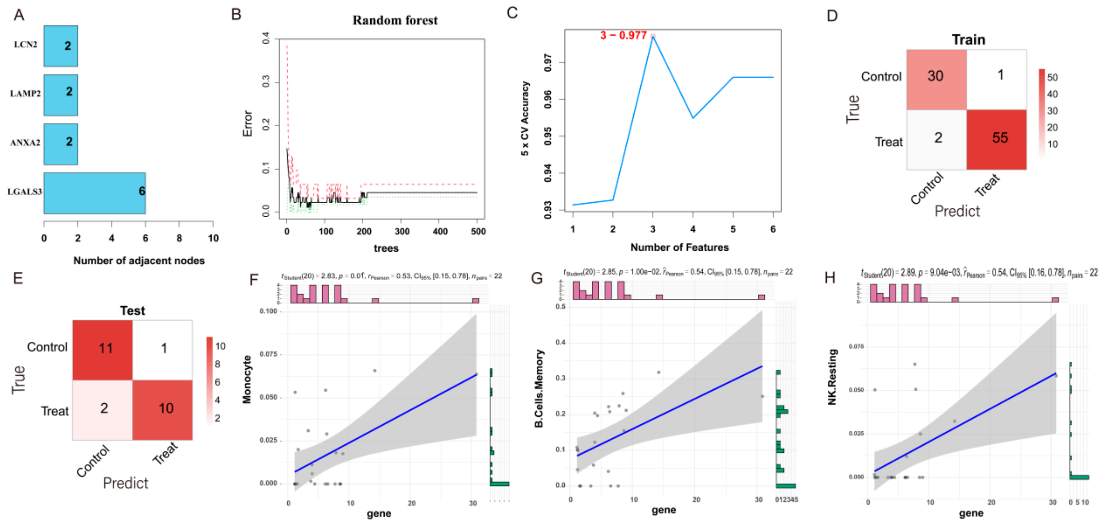

Figure S4. Identification of two gene biomarkers for HIBD and correlation analysis of core gene and three immune cells. (A)The number of adjacent nodes which the top 4 hub genes are connected. (B)The relationship between the number of RF tree and the model error. (C)3 genes are identified by SVM-RFE algorithm with the accuracy of 0.977.(D-E) The confusion matrix of the test set GSE184997 and GSE23333. (F-G) The correlation between LCN2 and 3 immune cells, including NK Resting, B Cells Memory, and Monocyte.

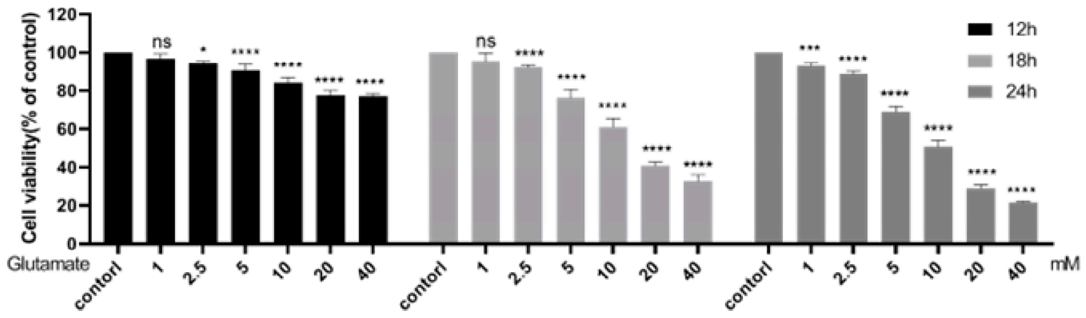

.

Figure S5. The effect of glutamate treatment on HT22 cells. The cells were treated with glutamate in different concentrations (1, 2.5, 5, 10, 20, and 40 mM) for 12, 18, and 24 h, then the cell viability of each group was measured using CCK-8.
